# SKILLED NURSING FACILITY VALUE-BASED PURCHASING PROGRAM: DISPARATE IMPACTS

**DOI:** 10.1093/geroni/igz038.243

**Published:** 2019-11-08

**Authors:** Jennifer L Gaudet Hefele, Joyce Wang, Emily Lim

**Affiliations:** 1 University of Massachusetts Boston, Massachusetts, United States; 2 University of Massachusetts Boston, Boston, Massachusetts, United States

## Abstract

In 2018, the federal government delivered the first round of incentive payments under the Skilled Nursing Facility (SNF) Value Based Purchasing (VBP) program. Incentive payments are based on post-acute readmissions performance. Facilities received payments that were either higher, on-par, or less-than typical. Our study sought to determine whether minority- and Medicaid-serving SNFs had the same likelihood of being among the top performers/incentive payment earners compared with SNFs serving more advantaged populations. We used publicly available data on SNF-VBP performance rankings available from CMS, linked to SNF characteristics publicly available from LTCFocus.org. Using logistic regression, our three analytic models examined the likelihood of being in the top 20% of SNF-VBP performance rankings among: SNFs in which the majority (>50%) of residents are African American(AA)/Black; SNFs in which the majority of residents are Hispanic/Latino; and SNFs in which 85% or more residents are Medicaid-pay. Results show that AA/Black-serving SNFs have two-thirds the odds of being in the top 20% of rankings compared to SNFs where the majority of residents are White (OR: 0.667 p<0.001). Hispanic/Latino-serving SNFs have less than half the odds (OR: 0.447 p<.01). High-Medicaid SNFs have less than one-third the odds of being in the top compared to SNFs with no Medicaid-pay residents (OR: 0.271 p<0.0001). Results are robust to alternate specifications of racial/ethnic and payer compositions. Lower payments to SNFs serving vulnerable populations is concerning, in that fewer resources may be available to these SNFs for patient care and quality improvement, which may result in creating or exacerbating disparities.

